# Supplementary material for: Chromothripsis during telomere crisis is independent of NHEJ, and consistent with a replicative origin
Source: Genome Res. 2019 May;29(5):737–49. doi: 10.1101/gr.240705.118 (PMC6499312; doi:10.1101/gr.240705.118)
Supplement: Supplemental Material [file supp_gr.240705.118_Supplemental_file_1.zip › contigs/annotated_contigs/DB46/contig.2.DB46_length_381_mean_cov_4.51181102362.docx]

**DB46_length_381_mean_cov_4.51181102362**

```
AACCCATACTGATGCCAGGCCTTATGTATATTATGATAGGACTAAAGTTGACTTTCTGTTTCTTTATGTTCATAGTCAACGTAGGCCAT
 >chr1:21987796-21988001 - E=5e-112
CATTCATTCATTGAACATTTATGGAGTGCTCACTAAAACAAGGAGACTATAGAGCTGATCAGGGTTGAGGGTCAGAGATTAGGTCATGA

GGCAGGGAGTCTCAGACACTGCTAAGC|AGCAGG|GCTGAGTTGGAACCCACTCTGTCCAGCCCTCTTGAGCCTGTGCCCTTCCTCACT
                                  >chr1:21986662-21986832 - E=7e-91
GGCTCACACTGCCCTGCTCTGTATCCAGGCTGTGTAGTTATGCATTTAGAGCCCTGGTTCCTGGAAGGCAGGGCTTCAGCCAAGCAACG

GGTTATTAAGCACCTACTGTGTGTCTT
```
